# Function of the large intestine and its interaction with the brain after ischemic stroke: a comprehensive literature review

**DOI:** 10.3389/fncel.2026.1735569

**Published:** 2026-03-12

**Authors:** Xuan Jia, Leticia Simo, Sydni Rosenfeld, Fengwu Li, Yingnan Zhou, Yuchuan Ding, Xiaokun Geng

**Affiliations:** 1 China-America Institute of Neuroscience, Beijing Luhe Hospital, Capital Medical University, Beijing, China; 2 Department of Neurology, Beijing Luhe Hospital, Capital Medical University, Beijing, China; 3 Department of Neurosurgery, Wayne State University School of Medicine, Detroit, MI, United States; 4 Neuro-Cardio-Vascular Diseases Research Lab, Xuanwu Hospital of Capital Medical University, Beijing, China

**Keywords:** gut-brain axis, intestinal immunity, large intestine, neuroinflammation, stroke

## Abstract

The large intestine, part of the distal gastrointestinal tract, is vital for water and electrolyte absorption and microbial fermentation. It is also a significant immune organ endowed with an extensive and intricate neural network. Intestinal epithelial cells are essential for endocrine regulation and maintaining the integrity of the intestinal barrier. Stroke, a leading cause of adult mortality and disability, occurs when there is a lack of oxygen to the brain and involves complex cerebrovascular dynamics that significantly impact systemic functions. In this framework, the gut-brain axis—the bidirectional circuitry connecting the gut and the central nervous system (CNS)—emerges as a critical interface. This review examines the immunological, neurological, endocrine, and barrier functions of the large intestine and explores its interplay with stroke pathophysiology. By detailing the interrelation between stroke and large intestinal functions, this paper aims to provide a foundational reference for advancing research into their intertwined mechanisms and identifying potential therapeutic targets.

## 1 Introduction

The large intestine plays a key role not only in water and electrolyte absorption, but also in immune regulation, neural signaling, endocrine activity, and maintenance of intestinal barrier integrity. Beyond these foundational roles, the large intestine is integral to immunity, neuroregulation, endocrine functions, and maintaining the homeostasis of the intestinal microenvironment.

Stroke is a neurological disorder characterized by the acute onset of focal or global neurological deficits resulting from either cerebral ischemia or intracranial hemorrhage. There are three subtypes of stroke: ischemic, hemorrhagic, and transient ischemic attacks. Ischemic strokes occur when a blood vessel that supplies blood to the brain is occluded whereas hemorrhagic strokes are due to the rupture of a blood vessel supplying the brain. Transient ischemic attacks, or mini-strokes, are strokes that cause a focal neurologic deficit lasting less than 24 h and are usually indicative of a higher risk of stroke occurring in the future (Grysiewicz et al., 2008).

According to the Global Burden of Disease Study 2021 (Collaborators GBDCoD, 2024), stroke accounts for a disproportionately high number of disability-adjusted life years (DALYs) compared with many other major diseases, underscoring its substantial global health burden. Ischemic strokes constitute over 80% of all stroke cases and are associated with considerable risks of mortality, recurrence, and long-term disability, resulting in a profound socioeconomic impact. In China—one of the countries with the largest number of stroke patients worldwide—stroke remains a leading cause of death and disability (Tu and Wang, 2023). Data from the Big Data Observatory Platform for Stroke of China indicate that among first-ever stroke patients, the in-hospital death rate was 1.9%, while the 12-month fatality, recurrence, and disability rates were 8.6%, 5.7%, and 16.6%, respectively (Tu et al., 2021). As socioeconomic development and lifestyle changes progress, the stroke burden in China is projected to rise substantially, with cerebrovascular events estimated to increase by approximately 50% by 2030 compared with 2010 (Wu et al., 2019). Stroke also imposes a heavy economic burden in China. A cost-of-illness study estimated that in 2018 the economic burden of stroke included ¥247.8 billion in direct costs and ¥704.4 billion in indirect costs (e.g., productivity losses due to premature mortality and disability) (Ma et al., 2024). These figures underscore the urgent need for effective secondary prevention strategies and integrated rehabilitation services to mitigate the growing personal, economic, and societal toll of stroke in China and globally (Tong et al., 2023; Qiao et al., 2024).

The gut-brain axis, a bidirectional communication network connecting the gastrointestinal tract and the brain, plays a pivotal role in stroke outcomes. Following a stroke, many patients develop gastrointestinal complications, including diarrhea, nausea, vomiting, gastrointestinal bleeding, and impaired intestinal barrier function. These issues can adversely affect prognosis and recovery (Camara-Lemarroy et al., 2014). Such observations underscore the critical link between gut function and stroke pathophysiology.

The present review aims to provide a comprehensive overview of the physiological functions of the large intestine and examine its interaction with stroke, shedding light on their interdependence and potential implications for therapeutic strategies.

This narrative review was based on a targeted literature search conducted primarily in the PubMed database. Relevant articles were identified using combinations of keywords related to the gut–brain axis, large intestine, stroke, intestinal immunity, enteric nervous system, gut microbiota, intestinal barrier, and endocrine regulation, corresponding to the thematic structure of each section. The search focused mainly on peer-reviewed articles published in English, with particular emphasis on studies from the past 10 years, while earlier landmark studies were included when necessary to provide essential background and mechanistic context. Both experimental and clinical studies relevant to the interaction between intestinal function and stroke were considered.

## 2 Functions of the large intestine

### 2.1 Intestinal immune system

#### 2.1.1 Distribution and function of large intestinal immune cells

The large intestine, as a key interface with the external environment, plays a critical role in immune defense, encountering pathogenic microorganisms and foreign antigens. Approximately 70% of the body's immune cells reside in the intestinal tract, encompassing both dispersed innate and adaptive effector cells and organized lymphatic tissue (Mowat and Agace, 2014). The intestinal mucosa, comprising the epithelium, underlying lamina propria, and muscularis mucosa, serves as the primary site of immune activity. The mucosal immune effector sites of the intestine include epithelium and lamina propria. Distinct populations of immune cells are present within both the epithelium and lamina propria, playing vital roles in immune processes (Rumbo and Schiffrin, 2005).

Within the colonic epithelium, intraepithelial lymphocytes (IELs) serve as first-line sentinels (Olivares-Villagomez and Van Kaer, 2018). In mice, IELs are broadly classified into type A (CD8αβ^+^ or CD4^+^ αβTCR^+^) and type B (γδTCR^+^ or CD8αα^+^ αβTCR^+^) subsets (Cheroutre et al., 2011). In humans, the colonic epithelium contains fewer IELs than the small intestine but is enriched in type A IELs, which exhibit cytotoxic and regulatory potential (Jabri and Ebert, 2007). These IELs maintain epithelial integrity, limit bacterial translocation, and rapidly respond to barrier disruption—functions that may be particularly relevant in systemic inflammatory states such as stroke (Olivares-Villagomez and Van Kaer, 2018).

In the lamina propria, CD4^+^ and CD8^+^ T cells dominate, with the colon and ileum harboring higher frequencies of interleukin-17 (IL-17)–producing CD4^+^ T (Th17) cells compared with the jejunum (Sathaliyawala et al., 2013). Although Th17 responses are essential for mucosal defense, they can become detrimental after stroke: activated colonic Th17 cells can enter the circulation and contribute to neuroinflammation, where IL-17A promotes neutrophil recruitment, blood–brain barrier disruption, and neuronal injury (Olivares-Villagomez and Van Kaer, 2018; Sathaliyawala et al., 2013).

Conversely, regulatory T cells (Tregs), which are also abundant in the colonic lamina propria, counterbalance excessive inflammation by secreting anti-inflammatory cytokines such as IL-10, thereby suppressing pathogenic Th17 responses and supporting neurological recovery (Mowat and Agace, 2014; Brandtzaeg, 2010). Plasma cells producing immunoglobulin A (IgA) constitute approximately 90% of all plasma cells in the colon. Secretory IgA neutralizes pathogens and toxins without inducing inflammation, reinforces epithelial barrier integrity, and limits microbial translocation—an important mechanism for restraining systemic inflammation that could exacerbate brain injury after stroke (Brandtzaeg, 2010).

Non-lymphoid innate immune cells, such as macrophages and dendritic cells (DCs), populate the gut-associated lymphoid tissue (GALT), where they play essential roles in antigen uptake and presentation (Gross et al., 2015). Other innate immune cells, including intestinal mast cells, are distributed throughout the gastrointestinal (GI) tract, primarily within the lamina propria and submucosa. These cells trigger inflammatory responses and modulate immune regulation by releasing mediators such as histamine (Yu and Perdue, 2001).

#### 2.1.2 Mucosal immune response

The innate immune system serves as the body's first line of defense against infections. Intestinal epithelial cells express pattern recognition receptors (PRRs), including extracellular Toll-like receptors (TLRs) and the intracellular NOD-like receptors (NLRs), which recognize pathogen-associated molecular patterns (PAMPs). For instance, TLR2 is predominantly expressed in epithelial cells of the proximal colon, with TLR4 and CD14 also present (Wang et al., 2010; Ortega-Cava et al., 2003). These receptors play pivotal roles in bacterial invasion of the intestinal mucosa. Upon activation by microbial penetration, PRRs initiate signaling cascades that recruit adaptor proteins and induce the production of inflammatory cytokines, chemotactic factors and antimicrobial molecules (Vaishnava et al., 2008; Akira, 2006; Zhao and Maynard, 2022).

The adaptive immune response in the intestine is predominantly initiated in the gut-associated lymphoid tissue (GALT), a specialized histological structure composed of Peyer's patches (PPs) in the small intestine, isolated lymphoid follicles (ILFs) in the large intestine, intraepithelial lymphocytes, and diffuse lymphocytes in the lamina propria. PPs and ILFs are enveloped by follicle-associated epithelium (FAE), which contains specialized antigen transporter cells known as M cells (Mowat and Agace, 2014). These M cells present antigens to DCs, which subsequently activate T and B lymphocytes, triggering adaptive immune responses (Filardy et al., 2023).

#### 2.1.3 Intestinal immunity and systemic immunity

The intestine is a pivotal organ in immune defense due to its constant exposure to external antigens and the abundance of immune cells within its structure. This exposure necessitates robust immune surveillance to distinguish between benign and harmful entities. Crosstalk between intestinal and systemic immunity is facilitated by the migration of immune cells and the transmission of signaling molecules, such as cytokines and chemokines, from the gut to other body systems (Mowat and Agace, 2014).

This interaction is essential for a cohesive immune response that can address local disturbances in the gut while also influencing systemic immune mechanisms (Probstel et al., 2020; Huang et al., 2020; Roy et al., 2021). Given the vast reservoir of immune cells in the gut, this continuous interaction between the intestines and external antigens, the GALT plays a crucial role in this dynamic, defending the intestinal mucosa against pathogens while shaping the systemic immune system (Huang et al., 2020; Vossenkamper et al., 2013). In systemic immunity, intestinal immunity is integral to maintaining homeostasis and defending against a spectrum of pathogens. The thymus is the major developmental site of peripheral T lymphocytes. Most IELs develop and mature from thymocytes and then migrate to the gut (Gangadharan et al., 2006). After antigen stimulation, T cells in GALT enter the blood circulation through lymph, and then return to the effector site of the intestinal mucosa, differentiate into effector cells, and play a local mucosal protective function. Gut-tropic integrins, a class of adhesion molecules, are involved in the homing process of immune cells to the intestine. In addition, this class of integrins is also able to enhance the peripheral migration ability of T cells, enabling them to cross immune barriers such as the blood-brain barrier (BBB). At present, pathogenic immune cells migrating from the intestine have been found in extraintestinal tissues, which have been confirmed to be related to a variety of extra-intestinal autoimmune diseases (Wu et al., 2024). B cells in the intestinal epithelium bind to antigens in the gut presented by M cells via the B cell receptor. The antigens found in the gut are mostly various strains of bacteria, so B cells continuously sample the intestinal microbiota. After sampling the antigens, the B cells, and other immune effector cells synthesize antibodies and various cytokines and chemokines that communicate with the systemic immune system. By mediating between immune tolerance and active defense, intestinal immunity emerges as a cornerstone of the body's overall immunological framework, warranting continued exploration in immunology (Ma et al., 2019).

### 2.2 Enteric nervous system

The enteric nervous system (ENS), the largest and most intricate division of the peripheral nervous system, comprises approximately 600 million neurons-more than those in the spinal cord. Often referred to as the “second brain”, this extensive network autonomously governs various gastrointestinal functions, including motor activities, sensory processing, nutrient absorption, and secretion, through the release of diverse neurotransmitters. The ENS operates in a highly integrated manner, ensuring efficient digestive processes and maintaining gastrointestinal homeostasis (Furness, 2012).

#### 2.2.1 Gut-brain axis

The gut-brain axis is a bidirectional communication network linking the central nervous system (CNS) and the ENS. It integrates neural, hormonal, and immunological signaling pathways, facilitating for extensive cross-talk between the brain and gastrointestinal tract. This axis is crucial in regulating digestion, mood, cognition and stress responses, underscoring the deep interconnection between gastrointestinal health and overall wellbeing (Agirman et al., 2021; Clemmensen et al., 2017; Hossain et al., 2024).

This communication enables feedback from the cortical and sensory centers in the brain to the gastrointestinal system and links external environmental inputs, such as nutrient sensing, with nervous system function. The vagus nerve, the tenth cranial nerve, mediates these interactions by transmitting signals between the brain and internal organs, including the gut. Composed of afferent and efferent neurons, the vagus nerve enables the brain to perceive the gut's environment and respond accordingly (Decarie-Spain et al., 2024).

Additionally, the gut-brain axis extends beyond basic CNS control of hunger and satiety. Alterations in gut microbiota or function can significantly influence cognitive function, social behavior, fear expression, and stress responses (Long-Smith et al., 2020). Gut hormones and neurotransmitters play a role in governing physiological processes like mood, cognition, and appetite control (Hong and Choi, 2024; Mandal et al., 2018; Holzer et al., 2012). For example, gut hormones such as Leptin and glucagon-like peptide-1 (GLP-1) act on the hypothalamus to regulate food intake, and also act on the hippocampus to affect hippocampus-dependent learning and memory (Gomez-Pinilla, 2008). Furthermore, animal studies suggest that leptin exerts antidepressant-like effects through central mechanisms by acting on leptin receptors expressed in mood-related brain regions, such as the hippocampus and hypothalamus, thereby supporting its role in mood regulation (Lu et al., 2006). Microbes in the gut produce metabolites that communicate with the brain. Among these metabolites are tryptophan precursors, various neurotransmitters, branched chain amino acids, lipopolysaccharide (LPS), short chain fatty acids, bile acids, and catecholamines. The interaction between microbe-derived metabolites and brain homeostasis may be implicated in neuropsychiatric problems (Suganya and Koo, 2020). Further, low-grade systemic inflammation is implicated in numerous neurological disorders, such as autism spectrum disorders, Parkinson's disease, Alzheimer's disease, and cerebrovascular conditions (Arteaga-Henriquez et al., 2022; Amor et al., 2010; Louka et al., 2022). All of these diseases have been linked with gut microbial dysbiosis, that is pathological changes in the gut microbiome. The translocation of microbes and their metabolites into the brain may provoke neuroinflammation which underlies the aforementioned neurological diseases (Suganya and Koo, 2020; Zou et al., 2023; Zhang et al., 2024; Kanuri and Sirrkay, 2024). Gut microbes can communicate bidirectionally with the brain through the pathways described above, further emphasizing the intricate and vital connection between the gut and the brain (Chakrabarti et al., 2022).

#### 2.2.2 Enteric neurons and neurotransmitters

Enteric neurons, the primary functional units of the ENS, regulate gastrointestinal motility, secretion, blood flow, and immune responses through a coordinated network of neurotransmitters, highlighting the functional complexity of the ENS in maintaining gut homeostasis (Michel et al., 2022; Sharkey and Mawe, 2023).

Within the ENS, distinct neurotransmitter systems exert complementary regulatory functions. Catecholamines, including noradrenaline and dopamine, predominantly mediate inhibitory control of intestinal motility, secretion, and vascular tone, while also modulating local immune responses. Cholinergic signaling serves as the principal excitatory pathway within the ENS, driving coordinated smooth muscle contraction and secretory activity. Serotonin (5-hydroxytryptamine, 5-HT), largely derived from enterochromaffin cells, plays a pivotal role in sensory transduction and the initiation of peristaltic reflexes. Vasoactive intestinal peptide (VIP) contributes to smooth muscle relaxation, epithelial secretion, and intestinal blood flow, thereby supporting mucosal homeostasis (Dicks, 2023).

Collectively, these neurotransmitter systems enable the ENS to function as an autonomous yet highly integrated interface linking gastrointestinal physiology to central nervous system regulation.

### 2.3 Endocrine function of the intestine

The intestine is the largest endocrine organ in the human body, with more than 30 gut hormone genes expressing over 100 bioactive peptides (Ahlman, 2001). A variety of endocrine cell types lining the walls of the gastrointestinal tract, collectively known as enteroendocrine cells (EECs), form the largest endocrine tissue in the body. These EECs sense and respond to changes in the gut's internal environment, primarily coordinating the digestive and absorptive functions of the gastrointestinal tract itself.

#### 2.3.1 Intestinal hormones

EECs are hormone-secreting cells located in the epithelial layer of the gastrointestinal tract. Most EECs are “open-type” cells, equipped with microvilli that directly interact with luminal contents, making them highly sensitive to chemical signals such as luminal food and pH (Lee et al., 2010; Ding et al., 2018). A smaller subset consists of “closed-type” cells that lack direct contact with the intestinal lumen. These cells respond to mechanical stimuli from gastrointestinal motility or regulation by other hormones and include enterochromaffin (EC) cells, enterochromaffin-like cells, D-cells, and P/D1-cells (Fakhry et al., 2019).

Despite accounting for only 1% of gastric and intestinal epithelial cells, EECs play a vital role in maintaining intestinal homeostasis by secreting diverse signaling molecules. Over 10 types of EECs have been identified, all functioning as sensory cells that influence metabolic and behavioral responses, such as insulin release and food consumption, in response to changes in intestinal contents (Gribble and Reimann, 2016).

EECs are classified by the hormones they produce. In the colon, the two primary types of EECs are Enteroendocrine L cells and EC cells (Jones et al., 2023). These cells can establish direct contact with gut lumen components, such as bacterial metabolites, through their apical surface. Their long lifespan allows them to integrate into the local signaling network, interacting with the enteric nervous system, glial cells, and immune cells in the submucosal layer.

##### Enteroendocrine L cells

L cells secrete GLP-1 and peptide tyrosine-tyrosine (PYY), potent appetite-suppressing hormones that regulate food intake. Receptors for these peptides are found in enteric neurons, vagus afferent fibers, and central nervous system regions such as the brainstem and the hypothalamus (De Silva and Bloom, 2012).

Enteroendocrine L cells, enriched in the distal ileum and colon, sense dietary nutrients and microbial metabolites to secrete glucagon-like peptide-1 (GLP-1) and peptide tyrosine tyrosine (PYY). In the proximal intestine, glucose is detected via SLC5A1 (SGLT1), leading to membrane depolarization and activation of voltage-gated Ca^2+^ channels. Long-chain fatty acids activate FFAR1/4 (GPR40/120), mobilizing intracellular Ca^2+^ through PLC–IP_3_ signaling, while monoacylglycerols engage the Gs-coupled receptor GPR119, increasing intracellular cAMP via PKA and Epac2 (Gribble and Reimann, 2016).

In the distal gut, where luminal nutrients are limited, L cells primarily respond to microbial metabolites. Short-chain fatty acids (SCFAs), including acetate, propionate, and butyrate, activate FFAR2/3 (GPR43/41) on colonic L cells to stimulate GLP-1 and PYY secretion. Notably, Lin et al. demonstrated that propionate and butyrate robustly induce GLP-1/PYY release even in Ffar3^−^/^−^ mice, suggesting the involvement of FFAR3-independent mechanisms, potentially including SCFA uptake through monocarboxylate transporters or epigenetic regulation (Lin et al., 2012). In addition, gut microbiota convert primary bile acids into secondary bile acids, which activate the Gs-coupled bile acid receptor TGR5 (GPBAR1) on L cells. Katsuma et al. showed that TGR5 activation increases intracellular cAMP and promotes GLP-1 secretion (Katsuma et al., 2005).

##### EC cells

EC cells are the primary source of serotonin production in the human body (Mawe and Hoffman, 2013). Serotonin activates various receptor families on gastrointestinal afferent nerve fibers, modulating gastrointestinal function.

#### 2.3.2 Targets of intestinal hormones

GLP-1 and PYY influence the satiety center, in the hypothalamus, suppressing appetite. This occurs either directly or indirectly via the vagal-brainstem-hypothalamus pathway. Research has revealed functional synaptic connections between enteroendocrine L cells, glial cells, and vagal afferent fibers. These connections enable precise and rapid signal transduction from the intestine to the brain while allowing the central nervous system to regulate L cells (Bohorquez et al., 2014, 2015).

While serotonin produced in the intestine cannot directly cross the blood-brain barrier (BBB), it affects gut-brain signaling by regulating vagal afferent fiber activity and intestinal inflammation. For instance, chemotherapy-induced nausea and vomiting illustrate gut-brain interaction, where excessive intestinal serotonin activates vagus nerve afferent fibers, leading to this response (Kennedy et al., 2017). The precursor of serotonin is tryptophan. Gut bacteria can also produce tryptophan and tryptamine which can directly affect central serotonin production. Further, certain bacterial strains can shape serotonergic signaling in the brain. It has been shown that specific microorganisms including *Akkermansia muciniphilia* can increase serotonin production in the hippocampus (Layunta et al., 2021). In addition, microbial metabolites short-chain fatty acids (SCFAs), which can be delivered into the circulation, increase brain serotonin concentrations (Sun et al., 2016). LPS, a major component of the outer membrane of Gram-negative bacteria, has been shown to reduce serotonin levels in the prefrontal cortex of mice (Zhu et al., 2015).

### 2.4 Function of the intestinal barrier

The gastrointestinal tract serves as the body's primary interface with the external environment, performing a dual role: absorbing essential nutrients into the bloodstream and acting as a barrier to prevent harmful substances, including microbes and antigens from entering the internal environment. The intestinal barrier comprises four interrelated components: the epithelial barrier, chemical barrier, immune barrier (GALT), and microbial barrier. The homeostasis of the intestinal microenvironment relies on the interactions among these barriers (Kurashima and Kiyono, 2017).

#### 2.4.1 Intestinal epithelial barrier

The intestinal epithelial barrier consists of a single layer of intestinal epithelial cells (IECs), including columnar epithelial cells, goblet cells, Paneth cells, enteroendocrine cells, tuft cells, and other cell types (Karam, 1999). These IECs originate from stem cells located near the base of intestinal crypts and are categorized as either absorptive or secretory enterocytes based on their functions (Noah et al., 2011).

##### Columnar epithelial cells

These absorptive cells, the most abundant in the intestinal epithelium, primarily facilitate nutrient absorption. Substances from the intestinal lumen can also penetrate the tissues through epithelial spaces, but tight junctions between epithelial cells serve as a critical safeguard. Tight junctions are composed of transmembrane proteins such as claudins, occludins, junctional adhesion molecule-A, and zonula occludens (Furuse et al., 1998, 1993; Martin-Padura et al., 1998; Citi et al., 1988). They are essential for maintaining intestinal barrier integrity preventing the translocation of bacteria and toxins (Allam-Ndoul et al., 2020). Damage to tight junctions can lead to systemic and intestinal inflammation (Turner, 2009), with increasing permeability linked to inflammatory bowel diseases (IBD) (Sayoc-Becerra et al., 2020).

#### 2.4.2 Intestinal chemical barrier

The chemical barrier includes secretions like mucus and antimicrobial proteins that create a protective layer over the intestinal epithelium.

##### Goblet cells and mucus production

Goblet cells secrete mucus (Birchenough et al., 2015), which forms a physical barrier between the intestinal lumen and the underlying epithelium. Mucus, primarily composed of MUC2, is the principal component of the mucus layer (Pelaseyed et al., 2014; Johansson et al., 2008). MUC2 forms a dense, net-like polymeric structure that requires calcium ions (Ca^2+^) for secretion. NLRP6 is a protein expressed in the intestinal epithelium that plays a role in recognizing damage-associated molecular patterns. Its function is involved in maintaining epithelial integrity and defense against pathogens (Venuprasad and Theiss, 2021). After NLRP6 is activated, the endoplasmic reticulum releases Ca^2+^. Mature MUC2 protein is transported into the intestinal lumen through vesicles (Liu et al., 2023). The mucus is structured into two layers: a loose outer layer, which serves as a habitat for the commensal bacteria, and a firm layer, which is impenetrable to bacteria. This two-layer system is prominent in the colon, whereas the small intestine has a single, loose mucus layer (Atuma et al., 2001). Penetration of the inner mucus layer by bacteria triggers inflammation, increasing the risk of conditions like ulcerative colitis, and colon cancer (Van der Sluis et al., 2006; Velcich et al., 2002).

##### Paneth cells and antimicrobial proteins

Paneth cells, found primarily in the distal small intestine, secrete antimicrobial proteins such as α-defensins, lysozyme, C-type lectins, and phospholipase A2 (PLA2). These substances inhibit the spread and colonization of pathogenic bacteria, reinforcing the chemical barrier (Lueschow and McElroy, 2020).

#### 2.4.3 Intestinal microbial barrier

The microbial barrier comprises the gut microbiota, a diverse community of microorganisms inhabiting the intestinal lumen. The microbiome not only promotes the digestion and absorption of nutrients but also improves resistance against harmful bacteria and pathogens. These resident microbiota form gut microbial barriers to maintain gut health. Microbiota participate in antimicrobial defense by releasing antimicrobial substances. These substances could occupy and block luminal wall attachment sites of bacterial colonization. This phenomenon has been described as “colonization resistance” (Morkl et al., 2020).

The human gut hosts approximately 10^14^ colony-forming units of bacteria, dominated by *Bacteroidetes* and *Firmicutes*, though composition varies between individuals (Qin et al., 2010). Disruption in the intestinal microbiota, such as increased bacterial translocation, can contribute to chronic intestinal inflammation (Natividad and Verdu, 2013). Prebiotics such as *Bifidobacteria* and *Lactobacilli* have shown promise in reducing pathogen infections and promoting gut health (Silva et al., 2004; Truusalu et al., 2008).

## 3 Interaction of the large intestine and brain in ischemic stroke

The bidirectional communication between the brain and the gut triggers a series of intestinal changes following a stroke, including dysbiosis of gut microbiota, immune imbalance, neuronal damage, and disruptions in endocrine regulation. These alterations can affect digestive absorption and impede recovery. Conversely, changes in gut function can influence stroke recovery and the development of complications.

### 3.1 Functional alterations in large intestine after stroke

**Figure 1:**
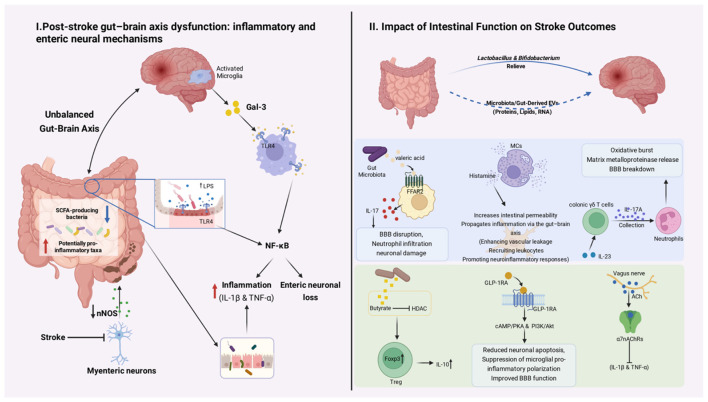
Bidirectional gut–brain axis alterations after ischemic stroke and their impact on stroke outcomes. Left panel: Post-stroke functional alterations in the large intestine and gut–brain axis dysfunction. Ischemic stroke induces microglial activation in the brain, leading to the release of inflammatory mediators such as galectin-3 (Gal-3), which can activate Toll-like receptor 4 (TLR4)–dependent signaling and NF-κB pathways. Concurrently, stroke disrupts colonic homeostasis, characterized by reduced short-chain fatty acid (SCFA)–producing bacteria and enrichment of potentially pro-inflammatory taxa, increased luminal lipopolysaccharide (LPS), intestinal barrier dysfunction, and impaired enteric neuronal signaling, including downregulation of neuronal nitric oxide synthase (nNOS). These changes collectively promote intestinal inflammation, enteric neuronal loss, and gastrointestinal dysmotility, reinforcing gut–brain axis imbalance after stroke. Right panel: Intestinal function influences stroke outcomes through microbiota-immune-neural pathways, including the valeric acid–FFAR2–IL-17 axis, mast cell–mediated histamine release, γδ T cell–driven neutrophil recruitment, and Treg/IL-10–dependent immune regulation, collectively shaping neuroinflammation, blood–brain barrier integrity, and neurological recovery after stroke. (Image created with BioRender.com, with permission.) Key mechanistic nodes highlighted include microbial metabolites (e.g., LPS, short-chain fatty acids, valeric acid), immune pathways (TLR4/NF-κB, IL-17, IL-10), enteric neuronal signaling (Gal-3/TLR4, nNOS), and barrier dysfunction, which are discussed in detail in Sections 3.1.1–3.1.5 and 3.2.1–3.2.3.

Before describing intestinal changes following stroke, it should be noted that colonic disorders present prior to stroke may also be associated with stroke risk and outcomes. Growing evidence suggests that chronic colonic disorders—particularly inflammatory bowel disease (IBD), including ulcerative colitis and Crohn's disease—are associated with an increased risk of ischemic stroke, independent of traditional vascular risk factors. A long-term nationwide cohort study reported that patients with IBD had an increased risk of stroke, especially ischemic events, and that the excess risk persisted for decades after diagnosis (Sun et al., 2023). Meta-analyses further support an overall elevated stroke risk in IBD populations (Fan et al., 2023). Clinically, IBD patients may experience acute ischemic stroke at a younger age and have higher rates of in-hospital complications, underscoring the cerebrovascular impact of chronic intestinal inflammation (Mui et al., 2025). Mechanistically, sustained systemic inflammation in IBD is proposed to drive endothelial dysfunction and a prothrombotic state, thereby promoting atherosclerosis and thrombosis and increasing the likelihood of cerebrovascular events (Khan and Azzam, 2025). This perspective provides a useful framework for the subsequent discussion of stroke-associated alterations in the large intestine (Figure 1).

#### 3.1.1 Gut microbiota

In the context of stroke, many of the gut microbiota alterations discussed here are derived from changes in the colonic microbiota, given its dominant role in short-chain fatty acid production and immune modulation.

Stroke patients often experience reduced physiological and immune functions, alongside swallowing dysfunction, leading to nutritional metabolism disorders and imbalances in gut microbiota. After acute stroke, reduced species diversity and bacterial overgrowth are observed, which contributes to intestinal barrier dysfunction and decreased intestinal motility (Singh et al., 2016). This disruption can result in constipation and a significant decline in beneficial bacteria such as *Bacteroides* and *Bifidobacterium* (Wang et al., 2021; Chang et al., 2021). Additionally, reduced levels of *Faecalibacterium, Streptococcus, Lactobacillus*, and *Oscillospira* were observed in the gut microbiome of monkeys post cerebral ischemia. Conversely, *Prevotella* was found to increase in abundance in the gut (Pluta et al., 2021). These changes in bacterial populations in the gut disrupt gut-brain axis homeostasis. The imbalance promotes the production of harmful metabolites like LPS, which induces inflammatory responses and exacerbates stroke-related injuries (Singh et al., 2016). Dysfunctional gut microbiota further increases the risk of post-stroke complications, including irritable bowel disease, and various autoimmune disorders (Pluta et al., 2021). The inflammation caused by gut dysbiosis post-stroke increases the number of lymphocytes which have been seen to migrate to the brain, leading to neuroinflammation (Singh et al., 2016). Neuroinflammation contributes to cognitive decline and depressive symptoms through multiple interconnected pathways. Pro-inflammatory cytokines can impair synaptic plasticity and disrupt long-term potentiation, leading to deficits in learning and memory. In parallel, neuroinflammatory activation alters large-scale brain network organization and functional connectivity, thereby compromising cognitive performance (Singh et al., 2016). Moreover, inflammatory signaling can influence neuroendocrine and monoaminergic systems, including HPA axis activation and neurotransmitter dysregulation, which has been implicated in the development of depressive symptoms (Wang et al., 2021). Notably, post-stroke cognitive impairment and depression are the most common neuropsychiatric disorders in stroke patients.

Emerging evidence indicates that gut dysbiosis contributes to post-stroke complications through four interrelated mechanisms: (1) Immune dysregulation: Depletion of short-chain fatty acid (SCFA)–producing commensals (e.g., *Faecalibacterium*) reduces colonic regulatory T cells (Tregs), weakening anti-inflammatory control and enabling systemic expansion of IL-17–producing γδ T cells that infiltrate the brain and amplify neuroinflammation (Piepke et al., 2021; Furusawa et al., 2013). (2) Metabolic disruption: Reduced microbial fermentation diminishes butyrate and other SCFAs, impairing epithelial energy metabolism and glucagon-like peptide-1 (GLP-1) secretion—thereby compromising both gut barrier integrity and neuroprotective hormone signaling (Lin et al., 2012; Sun et al., 2016). (3) Toxin barrier failure: Overgrowth of Gram-negative pathobionts increases luminal LPS, which translocates across a compromised intestinal barrier, triggering systemic endotoxemia and priming peripheral immune cells for exaggerated infiltration into the central nervous system (Singh et al., 2016; Stanley et al., 2016). (4) Neurotransmitter imbalance: Dysbiosis alters microbial synthesis of tryptophan, serotonin precursors, and γ-aminobutyric acid (GABA), disrupting central monoaminergic tone and vagal signaling—key pathways implicated in post-stroke depression and cognitive impairment (Layunta et al., 2021; Zhu et al., 2015). Collectively, these disruptions form a vicious cycle in which gut barrier breakdown permits bacterial translocation and systemic inflammation, aggravating brain injury; in turn, stroke-induced autonomic and neuroendocrine dysfunction further impairs gut motility, secretion, and immune homeostasis, perpetuating dysbiosis. This bidirectional deterioration underlies major post-stroke complications, including infection, cognitive impairment, depression, and gastrointestinal dysfunction, highlighting the gut microbiota as a central orchestrator of stroke outcomes.

#### 3.1.2 Intestinal immune system

In the acute phase of ischemic stroke, ischemic damage leads to the release of damage-associated molecular patterns (DAMPs) such as HMGB1, S100 proteins, and heat shock proteins (HSPs). These molecules initiate an innate immune response through Toll-like receptors (TLRs), triggering white blood cell infiltration and resident glial activation (Shi et al., 2019). Unlike pathogen-associated molecular patterns (PAMPs) from external sources, DAMPs originate internally and activate immune responses to address ischemic tissue damage. This ischemic injury increases integrins in white blood cells and adhesion molecules in endothelial cells (ECs), attracting leukocytes to the endothelium. Neutrophils, the first peripheral immune cell to infiltrate ischemic tissue, appear within hours and persist for up to a week post-injury (Stanzione et al., 2022). Other immune cells, such as monocytes, dendritic cells, natural killer (NK) cells, T and B lymphocytes, also cross the blood-brain barrier (BBB), amplifying the inflammatory response (Hernandez et al., 2023). In parallel with these central immune responses, ischemic stroke is also accompanied by profound alterations in intestinal immune homeostasis, including impaired mucosal immune regulation, skewed T cell responses, and increased intestinal permeability, which together may facilitate bacterial translocation and systemic inflammation.

#### 3.1.3 Enteric nervous system (ENS)

Stroke commonly induces gastrointestinal (GI) dysfunction—particularly impaired bowel motility—via a neurogenic gut mechanism driven by bidirectional brain–gut crosstalk. Two convergent pathways appear to be involved: Galectin-3 (Gal-3)/TLR4-mediated inflammation and impairment of nitrergic signaling. Following cerebral ischemia, activated microglia release Gal-3, a damage-associated molecular pattern that amplifies neuroinflammation through a TLR4/NF-κB signaling loop and contributes to blood–brain barrier disruption (Garcia-Revilla et al., 2022). Peripherally, elevated Gal-3 induces enteric neuronal loss in the colonic myenteric plexus in a TLR4-dependent manner (Cheng et al., 2016).

In addition, experimental evidence suggests that post-stroke bacterial translocation and LPS exposure can activate TLR4/NF-κB–associated inflammatory signaling and increase the expression of pro-inflammatory cytokines such as IL-1β and TNF-α (Xu et al., 2023). These inflammatory responses may further exacerbate intestinal inflammation and contribute to enteric nervous system (ENS) dysfunction. Concurrently, Kumar et al. demonstrated that stroke significantly downregulates neuronal nitric oxide synthase (nNOS) expression in myenteric neurons (Kumar et al., 2024). Because nitric oxide (NO) is essential for smooth muscle relaxation and coordinated peristalsis, reduced nitrergic signaling results in delayed gut transit and functional constipation, even in the absence of overt neuronal loss, indicating functional silencing of enteric neurons.

Together, Gal-3–driven inflammatory signaling and impaired nNOS-mediated neurotransmission disrupt gastrointestinal motility, promote dysbiosis and bacterial translocation, and may contribute to systemic inflammation that adversely affects neurological recovery. Targeting the Gal-3/TLR4 axis or preserving nitrergic function within the ENS may therefore represent promising strategies to mitigate post-stroke gastrointestinal complications and improve outcomes.

#### 3.1.4 Intestinal endocrine and epithelial barrier dysfunction

Stroke-induced disruptions in the hypothalamic-pituitary-adrenal axis, cholinergic dysfunction, and serotonin depletion are associated with post-stroke cognitive impairment and dementia (Zhou J. et al., 2023). Activation of enteric glial cells (EGCs) and subsequent intestinal inflammation damage the intestinal epithelial barrier, increasing permeability (Ye et al., 2021). Emerging evidence suggests that EGCs can sense microbiota-derived signals during barrier disruption, thereby amplifying local inflammatory responses. This dysfunction allows gut microbiota to translocate to systemic organs, potentially causing sepsis (Stanley et al., 2016) and exacerbating systemic inflammation, which impedes brain recovery (Zhou S. Y. et al., 2023). Notably, available evidence suggests that this barrier dysfunction is more closely related to impairment of the colonic epithelial barrier than to small intestinal absorptive defects, thereby facilitating bacterial translocation and systemic inflammation after stroke.

#### 3.1.5 Emerging pathways of gut–brain communication in stroke: extracellular vesicles and enteric glial sensing

Beyond classical neural, endocrine, and immune routes, extracellular vesicles (EVs)—including bacterial EVs (BEVs) derived from the gut microbiota—have emerged as important mediators of microbiota–host communication, carrying bioactive cargo that can influence host metabolism, immune signaling, and potentially distant organs along the gut–brain axis (Pirolli et al., 2021; Diaz-Garrido et al., 2021). Microbiota- or gut-derived EVs can carry proteins, lipids, and RNAs and modulate immune and metabolic signaling across organs (Haas-Neill and Forsythe, 2020). Although direct evidence for EV-mediated gut–brain communication in stroke remains limited, this mechanism is biologically plausible given stroke-associated barrier disruption and systemic inflammation.

Concurrently, enteric glial cells (EGCs) can sense microbial signals via pattern-recognition receptors, including TLRs, and respond by releasing mediators such as S100B and nitric oxide, thereby influencing inflammatory signaling and epithelial barrier function (Turco et al., 2014). Evidence for these processes largely derives from intestinal inflammation models, but they offer a plausible framework for how microbiota-derived signals may shape systemic inflammation and, indirectly, brain outcomes after stroke.

#### 3.1.6 Limitations of current evidence

Evidence for post-stroke colonic alterations is predominantly derived from animal models, which differ from humans in intestinal anatomy, immune composition, and microbiota structure. Experimental conditions such as antibiotic exposure, dietary control, and stress responses may further confound interpretation. In addition, human studies remain largely observational, limiting causal inference regarding the contribution of colonic dysfunction to stroke outcomes.

To facilitate integration of the mechanisms discussed above, a summary table outlining key gut–brain axis pathways involved in stroke is provided in Table 1.

**Table 1:** Gut–brain axis mechanisms linking large intestinal function to stroke outcomes.

**Axis level**	**Key components**	**Representative findings**	**Model/population**	**Stroke relevance**	**Evidence level**
Gut microbiota	*Faecalibacterium, Bifidobacterium, Lactobacillus, Prevotella*	Stroke induces gut dysbiosis characterized by loss of SCFA-producing commensals and expansion of pathobionts, which correlates with neuroinflammation and worse functional outcomes	MCAO mice; non-human primates; AIS patients	Neuroinflammation, cognitive impairment, post-stroke complications	Preclinical + observational
Microbial metabolites	SCFAs (butyrate, propionate); valeric acid; LPS	Reduced SCFAs impair epithelial energy metabolism and GLP-1 secretion, whereas valeric acid activates FFAR2 and drives IL-17–mediated neuroinflammation; LPS translocation promotes systemic inflammation	Aged mice; ischemic stroke models; human MR analysis	BBB disruption, immune activation, neurological deficits	Preclinical + genetic causal inference
Intestinal immunity	γδ T cells; Tregs; IL-17A; IL-10; mast cells	Stroke-induced dysbiosis skews gut immunity toward IL-17A-producing γδ T cells and mast cell activation, while Treg/IL-10 signaling exerts protective effects	MCAO mice; immune knockout models	Neutrophil recruitment, infarct expansion, inflammation control	Preclinical
ENS dysfunction	Galectin-3; TLR4; nNOS	Stroke triggers Gal-3/TLR4-mediated enteric neuroinflammation and downregulation of nNOS, leading to impaired gut motility and bacterial translocation	MCAO mice	Constipation, dysbiosis, systemic inflammation	Preclinical
Endocrine signaling	GLP-1; vagus nerve; VIP	Disrupted gut hormone signaling and vagal dysfunction contribute to neuroinflammation, while GLP-1 and VIP exhibit neuroprotective and anti-inflammatory effects	Rodent stroke models; pilot clinical studies	Neuroprotection, BBB integrity, recovery	Preclinical + early clinical
Gut-targeted interventions	Probiotics; prebiotics; FMT; VNS; GLP-1 RAs	Modulating gut microbiota or gut–brain signaling improves neurological outcomes and reduces inflammation in experimental stroke; clinical evidence remains limited	Animal models; small human studies	Functional recovery, infection reduction	Preclinical dominant

AIS, acute ischemic stroke; MCAO, middle cerebral artery occlusion; SCFAs, short-chain fatty acids; LPS, lipopolysaccharide; FFAR2, free fatty acid receptor 2; MR, Mendelian randomization; Tregs, regulatory T cells; nNOS, neuronal nitric oxide synthase; GLP-1, glucagon-like peptide-1; VIP, vasoactive intestinal peptide; FMT, fecal microbiota transplantation; VNS, vagus nerve stimulation; BBB, blood–brain barrier.

Evidence level definition: preclinical refers to animal or mechanistic studies; observational refers to human associative studies; genetic causal inference refers to Mendelian randomization analyses; early clinical refers to pilot or small-scale interventional studies.

### 3.2 Impact of intestinal function on stroke outcomes

To facilitate mechanistic clarity, the major gut-derived factors influencing stroke outcomes can be categorized into defined nodes, including specific microbial taxa and metabolites (e.g., valeric acid, butyrate, LPS), their target immune or neural cell populations (γδ T cells, Tregs, mast cells, enteric neurons), and downstream signaling pathways (FFAR2/IL-17, HDAC/Foxp3/IL-10, TLR4/NF-κB). These nodes are summarized in Figure 1 and further elaborated in the following sections.

#### 3.2.1 Gut microbiota and recovery

The gut microbiota significantly influences stroke susceptibility and recovery, with age-related microbial shifts playing a causal role in post-stroke neuroinflammation and outcomes. Spychala et al. demonstrated that fecal microbiota transplantation (FMT) from young to aged mice improved stroke recovery, whereas transfer of aged microbiota into young mice worsened brain injury, highlighting microbiota composition—rather than chronological age—as a key determinant of cerebrovascular resilience (Spychala et al., 2018).

This effect is mechanistically linked to the gut microbiota–valeric acid–FFAR2–IL-17 axis. Zeng et al. reported that aged mice harbor a dysbiotic microbiome enriched in valeric acid–producing bacteria (Zeng et al., 2023). Unlike anti-inflammatory short-chain fatty acids such as butyrate, the microbial metabolite valeric acid activates free fatty acid receptor 2 (FFAR2/GPR43) on immune cells, driving IL-17 production—a cytokine that promotes blood–brain barrier disruption, neutrophil infiltration, and neuronal damage after stroke. Elevated valeric acid and IL-17 levels correlated with worse neurological deficits, and genetic or antibiotic ablation of this pathway abolished its detrimental effects (Zeng et al., 2023).

Consistent with these preclinical findings, human observational studies suggest that gut microbiota dysbiosis is associated with functional outcomes in acute ischemic stroke patients (Chang et al., 2021), and Mendelian randomization analyses further support a causal relationship between gut microbiota composition, microbiota-derived metabolites, and stroke risk in humans (Wang Q. et al., 2023). Specifically, multiple microbial taxa were identified as being causally associated with stroke susceptibility, while stroke itself altered the abundance of several taxa, including *Bifidobacteriaceae* and *Desulfovibrio*, whose genetically predicted abundance was associated with lower stroke risk. However, clinical interventional trials evaluating FMT in stroke patients are still lacking, and further studies are required to establish its safety and efficacy in humans.

Together, these findings indicate that age-associated dysbiosis exacerbates stroke outcomes via a pro-inflammatory valeric acid/FFAR2/IL-17 cascade, whereas microbiome-targeted interventions hold therapeutic potential. In experimental models, probiotic supplementation with strains such as *Lactobacillus* and *Bifidobacterium* has been shown to improve neurological deficits, reduce neuroinflammation, and mitigate post-stroke complications (Lee et al., 2020; Wang Y. et al., 2023). Collectively, these data highlight the translational relevance of microbiota-derived mediators and suggest that targeting the gut microbiota may represent a promising strategy to improve stroke recovery, particularly in older individuals.

#### 3.2.2 Immune contributions to stroke outcomes

The gut, housing approximately 70% of the body's immune cells, acts as the largest immune organ and a key mediator of systemic and neuroinflammatory responses after stroke (Vighi et al., 2008). Early after cerebral ischemia, gut mast cells (MCs) become activated—amplified by sympathetic overdrive and intestinal barrier disruption—resulting in increased histamine release. Histamine increases intestinal permeability and propagates inflammation via the gut–brain axis by enhancing vascular leakage, recruiting leukocytes, and promoting neuroinflammatory responses, including microglial activation (Conesa et al., 2023; Blasco et al., 2020; Benakis et al., 2016; Bulfone-Paus et al., 2017). MC stabilizers, such as cromolyn, reduce histamine release, attenuate inflammation, and improve stroke outcomes (Conesa et al., 2023; Blasco et al., 2020; Strbian et al., 2007).

Concurrently, colonic γδ T cells—shaped by commensal microbiota—are rapidly activated after stroke and produce IL-17A in response to IL-23 (Benakis et al., 2016). IL-17A recruits neutrophils to the brain, exacerbating injury through oxidative burst, matrix metalloproteinase release, and blood–brain barrier breakdown (Nielsen et al., 2017; Li et al., 2023; Gelderblom et al., 2012). Neutralization of IL-17A or depletion of γδ T cells reduces infarct size and improves neurological recovery (Benakis et al., 2016; Gelderblom et al., 2012). In contrast, IL-10—primarily derived from microbiota-induced regulatory T cells (Tregs)—suppresses IL-17A signaling and limits neutrophil infiltration (Piepke et al., 2021; Suo et al., 2023). In mice, colonic Tregs are abundant and can be expanded by Clostridia-derived butyrate through histone deacetylase inhibition and Foxp3 upregulation, thereby enhancing IL-10 production and immune tolerance (Furusawa et al., 2013). Higher IL-10 levels are associated with improved stroke outcomes (Piepke et al., 2021; Suo et al., 2023).

Collectively, post-stroke immunity is governed by a tripartite gut immune network comprising MC-driven histamine release, γδ T cell–IL-17A–mediated neutrophil amplification of injury, and Treg/IL-10–dependent immune restraint. Targeting these pathways may offer promising strategies to modulate gut–brain crosstalk and enhance post-stroke recovery.

#### 3.2.3 ENS and endocrine interventions

The vagus nerve represents a critical neuroimmune conduit linking the enteric nervous system (ENS) with the central nervous system and has emerged as a promising therapeutic target in stroke. Beyond its role in autonomic regulation, vagal signaling exerts potent anti-inflammatory effects through the cholinergic anti-inflammatory pathway, primarily mediated by α7 nicotinic acetylcholine receptors (α7nAChRs) expressed on immune cells (Li et al., 2022; Yang J. et al., 2022; Yang X. et al., 2022). Activation of this pathway suppresses pro-inflammatory cytokines such as TNF-α and IL-1β, attenuates peripheral immune activation, and limits secondary neuroinflammation after cerebral ischemia.

Consistent with these mechanisms, vagus nerve stimulation (VNS) improves neurological outcomes and reduces infarct volume in experimental models of ischemic stroke (Khodaparast et al., 2016), and early clinical studies suggest its feasibility and potential efficacy in stroke patients (Wu et al., 2020). By dampening systemic inflammation and modulating microglial activation, VNS may indirectly preserve blood–brain barrier integrity and promote post-stroke recovery.

In parallel, glucagon-like peptide-1 receptor agonists (GLP-1 RAs), widely used in type 2 diabetes, have demonstrated neuroprotective effects in stroke models. These benefits are mediated not only through metabolic regulation but also via direct central actions involving cAMP/PKA and PI3K/Akt signaling pathways, leading to reduced neuronal apoptosis, suppression of microglial pro-inflammatory polarization, and improved blood–brain barrier function (Merchenthaler et al., 1999; Zhang et al., 2015; Sattar et al., 2021; Abdel-Latif et al., 2018; Teramoto et al., 2011; Mi et al., 2024). However, further clinical studies are required to establish their therapeutic efficacy in stroke patients (Larsson et al., 2019; Zhao et al., 2024).

VIP, expressed in both the central nervous system and the intestinal submucosal plexus (Lelievre et al., 2007), exerts anti-inflammatory and neurotrophic effects. In experimental stroke models, VIP reduces brain injury and enhances neurogenesis, potentially through vascular endothelial growth factor–dependent angiogenesis and immunomodulatory actions (Yang et al., 2015; Pascal et al., 2022). Nevertheless, whether gut-derived VIP contributes to stroke recovery via gut–brain signaling remains to be fully elucidated.

Finally, enteric glial cells (EGCs) exhibit neurogenic and immunoregulatory properties and may influence ENS integrity and gut–brain communication after stroke. Emerging evidence suggests that EGCs could serve as a potential cell source for regenerative or transplantation-based strategies aimed at promoting neural repair during stroke recovery (Hacene et al., 2022).

#### 3.2.4 Limitations of current evidence

Evidence linking intestinal function to stroke outcomes is largely based on preclinical studies and associative human data. While animal experiments provide mechanistic insights into microbiota–immune–brain interactions, confounding factors such as antibiotic exposure, nutritional status, stress responses, and swallowing dysfunction are difficult to control and may influence outcomes. Human observational and Mendelian randomization studies support associations but cannot fully establish causality at the interventional level. Well-designed clinical trials are required to determine whether targeting intestinal function can meaningfully improve stroke recovery.

### 3.3 Emerging therapeutic strategies targeting the gut–brain axis in stroke

**Figure 2:**
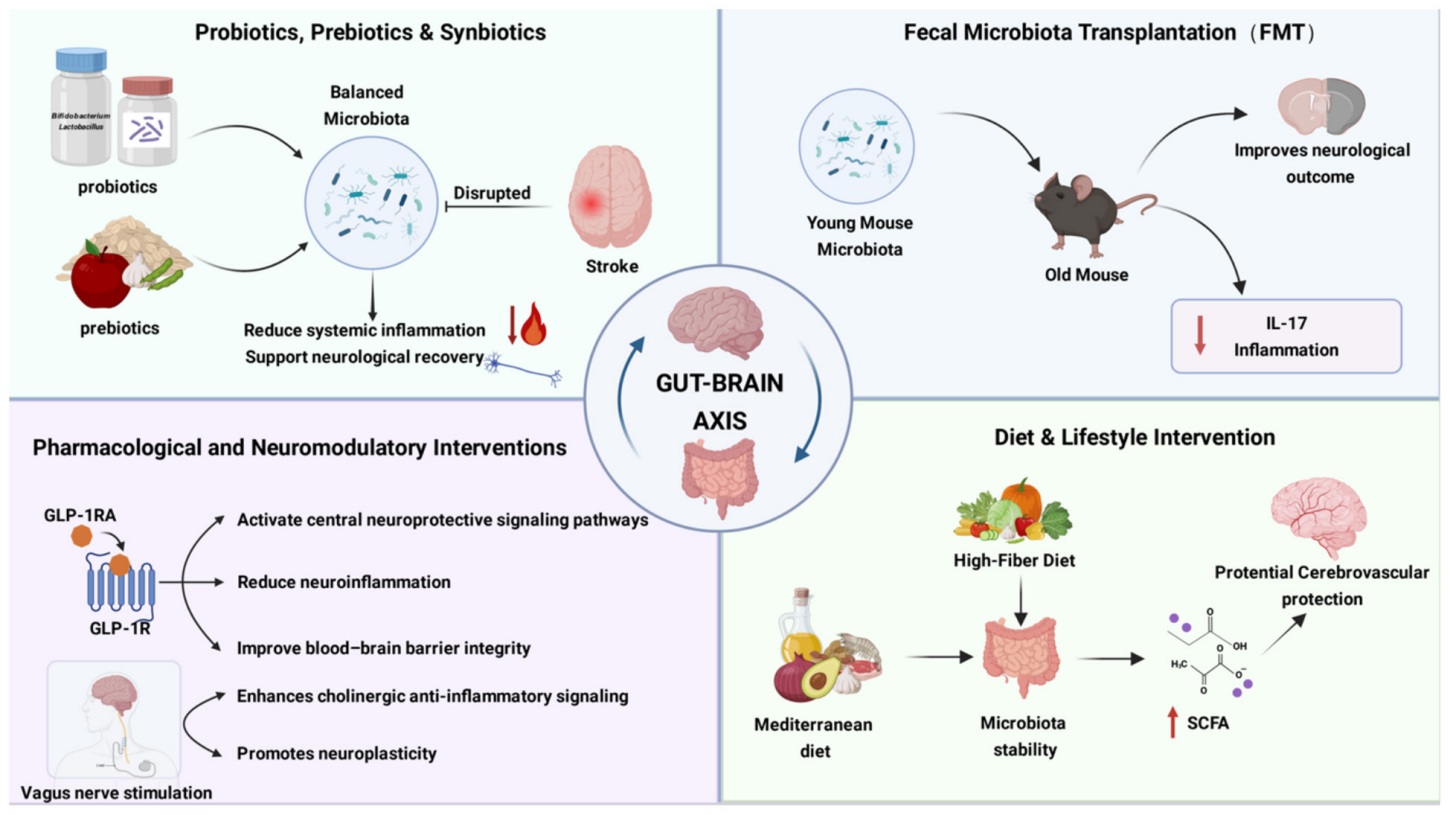
Gut-targeted interventions modulating stroke outcomes via the gut–brain axis. This schematic summarizes representative therapeutic strategies that influence stroke outcomes by targeting intestinal function and gut–brain communication. Probiotics, prebiotics, and synbiotics may help restore microbiota balance, reduce systemic inflammation, and support neurological recovery. Fecal microbiota transplantation (FMT) studies in animal models demonstrate that transplantation of young donor microbiota into aged recipients improves neurological outcomes and reduces IL-17–mediated inflammation. Pharmacological and neuromodulatory interventions, including glucagon-like peptide-1 receptor agonists (GLP-1 RAs) and vagus nerve stimulation (VNS), exert neuroprotective effects by attenuating neuroinflammation, improving blood–brain barrier integrity, enhancing cholinergic anti-inflammatory signaling, and promoting neuroplasticity. Dietary and lifestyle interventions, such as high-fiber and Mediterranean-style diets, contribute to microbiota stability and increased short-chain fatty acid (SCFA) production, which may confer potential cerebrovascular protection. (Image created with BioRender.com, with permission).

Recent advances in understanding gut–brain axis mechanisms have facilitated the development of therapeutic strategies targeting intestinal homeostasis to improve stroke outcomes. These approaches aim to modulate gut microbiota composition, reinforce barrier integrity, attenuate systemic inflammation, and enhance neuroprotective signaling (Figure 2).

#### 3.3.1 Probiotics, prebiotics, and synbiotics

Both preclinical and clinical studies suggest that probiotic supplementation, particularly with Lactobacillus and Bifidobacterium strains, can mitigate post-stroke gut dysbiosis, reduce systemic inflammation, and improve neurological recovery. In rodent models, probiotics decrease circulating pro-inflammatory mediators and promote functional recovery, while a randomized controlled trial in acute ischemic stroke patients reported reduced infection rates and shorter hospital stays following multi-strain probiotic treatment (Lee et al., 2020; Wang Y. et al., 2023). Prebiotics such as inulin enhance the growth of short-chain fatty acid–producing bacteria, whereas synbiotic formulations may exert synergistic effects on microbiota restoration and barrier function.

#### 3.3.2 Fecal microbiota transplantation (FMT)

FMT has emerged as a potent experimental strategy to restore microbial diversity after stroke. In aged mouse models, transplantation of microbiota from young donors reduced infarct volume, suppressed IL-17–mediated neuroinflammation, and improved functional recovery (Spychala et al., 2018; Zeng et al., 2023).

While direct interventional trials of FMT in stroke patients are currently lacking, human observational studies and Mendelian randomization analyses indicate that gut microbiota composition and microbiota-derived metabolites are causally associated with stroke risk and functional outcomes (Chang et al., 2021; Wang Q. et al., 2023), supporting the translational relevance of microbiota-targeted interventions.

#### 3.3.3 Pharmacological and neuromodulatory interventions

Several clinically approved therapies exert neuroprotective effects partly through gut–brain axis modulation. Glucagon-like peptide-1 receptor agonists activate central neuroprotective signaling pathways, reduce neuroinflammation, and improve blood–brain barrier integrity in experimental stroke models (Teramoto et al., 2011; Mi et al., 2024).

In parallel, vagus nerve stimulation enhances cholinergic anti-inflammatory signaling and promotes neuroplasticity, with early clinical trials demonstrating functional benefits when combined with rehabilitation (Wu et al., 2020).

#### 3.3.4 Dietary and lifestyle interventions

Dietary strategies, including high-fiber and Mediterranean-style diets, promote a resilient gut microbiome and increase short-chain fatty acid production, which may confer long-term cerebrovascular protection. Conversely, Western dietary patterns exacerbate dysbiosis and endotoxemia, highlighting diet as a modifiable contributor to stroke susceptibility and recovery (Long-Smith et al., 2020).

#### 3.3.5 Limitations of current evidence

Gut-targeted therapeutic strategies for stroke are largely supported by preclinical data, with limited validation in randomized clinical trials. Variability in intervention timing, formulation, dosage, and patient selection complicates comparison across studies. Importantly, the safety, durability, and patient-specific efficacy of microbiota-based interventions remain to be systematically evaluated.

### 3.4 Gut-lung interaction in stroke

The lymphatic vessels in the gut serve as a bridge between the gut and distant organs, including the lungs. Mesenteric lymph bypasses portal circulation, allowing cytotoxic factors to travel directly through the pulmonary circulation, potentially causing acute lung injury. This phenomenon is referred to as the *gut-lymph-lung axis* (Deitch, 2010). Metabolites produced by the gut microbiome, such as short-chain fatty acids (SCFAs), are distributed via the portal system to various organs, including the lungs, where they can exert both beneficial and harmful effects (Ney et al., 2023).

Dysregulation of the intestinal microbiota can lead to an increase in LPS levels, which exacerbate immune-mediated lung injury. Conversely, LPS-induced lung injury can trigger low-grade intestinal inflammation during acute injury phases (Xu et al., 2021). One of the most common complications following ischemic stroke is post-stroke pneumonia. Structural disruption of the gut and increased barrier permeability after stroke facilitate the translocation of gut bacteria to the lungs, thereby inducing pulmonary infections (Wen et al., 2019). Consistent with the observations discussed above, gut–lung interactions after stroke may be influenced in part by colonic barrier disruption and microbiota translocation.

#### Limitations of current evidence

Current evidence linking gut–lung interactions to stroke complications is limited and primarily inferential. Most studies focus on systemic inflammation rather than direct mechanistic pathways, underscoring the need for integrative human studies.

## 4 Perspective and prospective

The stability of intestinal function and the maintenance of microenvironmental homeostasis rely on the intricate balance of the intestinal immune system, the enteric nervous system, enteroendocrine functions, barrier integrity, and the gut microbiota. The bidirectional interactions of the “gut-brain axis” have become central to understanding the interplay between gastrointestinal systems and CNS disorders, positioning this axis as a key focus of modern neurological research.

Stroke disrupts intestinal function, leading to imbalances in the gut microenvironment that can exacerbate CNS injuries and contribute to stroke-related complications. Conversely, strategies aimed at regulating intestinal homeostasis may alleviate post-stroke injuries, highlighting the therapeutic potential of gut-targeted interventions in stroke recovery. The gut microbiota plays a pivotal role in this dynamic by influencing the CNS through immune modulation, neural signaling, and hormone secretion.

While current studies have provided critical insights, substantial gaps remain in our understanding of how stroke affects specific functions of the large intestine. In particular, future research should focus on elucidating the mechanisms by which stroke-induced alterations in gut microbiota influence neuroinflammation, neurotransmission, and neuroendocrine pathways during cerebrovascular events.

In addition, there is a significant opportunity to develop gut-targeted interventional strategies to enhance stroke rehabilitation outcomes. Priority areas include evaluating dietary modulation, prebiotics, probiotics, and synbiotic approaches in well-designed experimental and clinical studies. Long-term observational studies are also needed to clarify the chronic effects of stroke on intestinal function and the reciprocal impact of gut health on central nervous system recovery. Together, these focused research directions may facilitate the identification of novel therapeutic targets and support the translation of gut–brain axis–based interventions into stroke recovery.
